# Causal Effect of Serum Magnesium on Osteoporosis and Cardiometabolic Diseases

**DOI:** 10.3389/fnut.2021.738000

**Published:** 2021-12-03

**Authors:** Bin He, Liang Xia, Jinqiu Zhao, Lifeng Yin, Muzi Zhang, Zhengxue Quan, Yunsheng Ou, Wei Huang

**Affiliations:** ^1^Department of Orthopedics, The First Affiliated Hospital of Chongqing Medical University, Chongqing, China; ^2^Department of Surgical Intensive Care Unit, The First Affiliated Hospital of Chongqing Medical University, Chongqing, China; ^3^Department of Infectious Diseases, The First Affiliated Hospital of Chongqing Medical University, Chongqing, China

**Keywords:** serum magnesium, lumbar spine BMD, osteoporosis, cardiometabolic diseases, Mendelian randomization study

## Abstract

Serum magnesium is associated with osteoporosis and cardiometabolic diseases, but their causal associations remain elusive. We used the two-sample Mendelian randomization (MR) study to explore the causal roles of serum magnesium on osteoporosis and cardiometabolic diseases by using the aggregated genome-wide association studies (GWASs). Six single-nucleotide polymorphisms (SNPs, *p* < 5 × 10^−8^) associated with serum magnesium concentrations were all used as instrumental variables. A genetic predisposition to higher serum magnesium concentrations was inversely associated with lower lumbar spine bone mineral density (BMD, beta-estimate: −1.982, 95% CI: −3.328 to −0.635, SE: 0.687, *p* = 0.004), which was further confirmed by multiple sensitivity analyses. There was limited evidence of associations between serum magnesium and type 2 diabetes, coronary artery disease, heart failure, and atrial fibrillation. This work provided strong evidence that genetically increased serum magnesium concentrations were causally associated with low lumbar spine BMD and suggested that serum magnesium concentrations may be crucial to prevent osteoporosis.

## Introduction

Magnesium, the second most abundant intracellular cation, participates in many physiological processes in osteoporosis and cardiovascular function, such as vascular tone, endothelial function, glucose, and insulin metabolism (1–3). Experimental studies reported that magnesium insufficiency aggravated inflammatory bone resorption and promoted atherosclerosis (4–6). Previous studies revealed the benefits of magnesium supplementation to improve endothelial function (7, 8) and reduce blood pressure (9, 10), arterial stiffness (11), and postoperative arrhythmias (12, 13). However, there is no evidence from randomized controlled trials assessing the causal effect of serum magnesium on osteoporosis or cardiometabolic diseases.

Chronic inflammation has a strong association with the concentration of magnesium and osteoporosis (14–17). The pathogenesis of cardiovascular diseases (e.g., coronary artery disease, heart failure, and atrial fibrillation) and type 2 diabetes are also affected by chronic inflammation (18–21). Osteoporosis and cardiometabolic diseases have a robust connection with pathogenesis, and thus this study aims to study the influence of serum magnesium concentrations for the prevention and treatment of osteoporosis and cardiometabolic diseases.

Observational studies documented that high serum magnesium concentrations might be associated with a modest reduction in osteoporosis and cardiovascular diseases, such as coronary heart disease, but it was elusive whether these associations were causal (1, 22, 23). These observational studies may be subject to confounding and reverse causality, which may affect the association between serum magnesium and osteoporosis/cardiometabolic diseases. Genome-wide association studies (GWASs) demonstrated that osteoporosis and cardiometabolic diseases were highly polygenic traits (24–29), and thus this two-sample Mendelian randomization (MR) study is intended to explore the causal relationship between serum magnesium and osteoporosis/cardiometabolic diseases, which benefits to prevent and treat these diseases.

## Methods

### Genetic Instrument for Serum Magnesium

The largest available GWAS associated with serum magnesium concentrations reported the joint analysis of the discovery (*n* = 15,366 individuals) and replication (*n* = 8,463 individuals) cohorts (30). Serum magnesium concentrations were measured using the method described by Gindler and Heth with metallochromic dye, Calmigate [1,-[1-hydroxy-4-methyl-2-phenylazo)-2-napthol-4-sul-fonic acid] in the Atherosclerosis Risk in Communities (ARIC) study, by METPATH in Framingham Heart Study (FHS) with a Merck Diagnostica kit (method Xylidyl blue) on an Elan Autoanalyzer (Merk) in Rotterdam Study (RS), with a Xylidylblue kit on a Modular analyzer (Roche) in the Cooperative Health Research in the Region of Augsburg (KORA) study, or using a commercial colorimetric test (Roche Diagnostics, Mannheim, Germany) with a Hitachi 717 autoanalyzer in the Study of Health in Pomerania (SHIP) study. The measurement unit was mmol/L. All tests were adjusted for age, sex, and study center.

All single-nucleotide polymorphisms (SNPs) that reached genome-wide significance (*p* < 5 × 10^−8^) were selected as the instrumental variables. Then, six SNPs were initially found to serve as instrumental variables. We measured linkage disequilibrium (LD, *R*^2^ <0.001, window size = 10,000 kb) between selected SNPs using European samples from the 1,000 genomes project, and no SNP was excluded due to strong LD. Six SNPs were finally used as instrumental variables (Supplementary Table 1). If SNPs were unavailable in the outcome dataset, the proxy SNPs in high linkage disequilibrium (LD, *r*^2^ > 0.9) with the closest distance to the original SNPs were used as the instrumental variables. The heat map showed the effects (beta and SE) of instrumental variables associated with serum magnesium (Supplementary Figure 1).

Prior to any MR analysis, we first performed a standard allele harmonization to align alleles on the forward strand, and palindromic SNPs were aligned when minor allele frequencies (MAFs) were <0.3 or were otherwise excluded. The effect of ambiguous SNPs with non-concordant alleles (e.g., A/G vs. A/C) and palindromic SNPs with an ambiguous strand (i.e., A/T or G/C) was corrected, or the ambiguous and palindromic SNPs were directly excluded from the above-selected instrument SNPs in harmonizing process.

### Outcome Data Sources

Summary-level data for the genetic associations with the outcomes were obtained from several largest GWASs (Table 1). Briefly, for osteoporosis, we used data involving forearm BMD, femoral neck BMD, and lumbar spine BMD (measured by dual X-ray absorptiometry) among 53,236 individuals (31), heel BMD (estimated by heel quantitative ultrasound), and fracture (excluding fracture cases including the fracture of the skull, face, hands, feet, and pathological fractures due to malignancy, atypical femoral fractures, periprosthetic, and healed fracture) among 4,26,824 people (32). In terms of cardiometabolic diseases, the outcome measures included type 2 diabetes among 8,98,130 subjects (33), coronary artery disease among 5,47,261 individuals (34), heart failure among 9,77,323 people (35), and atrial fibrillation in 5,87,446 persons (36). Fasting glucose, fasting insulin, and HbA1c were included to assess the glycaemic traits in the large-scale GWAS among 2,81,416 individuals (37). Most GWASs were adjusted for sex, BMI, and genetic principal components. Summary statistics for the SNPs related to each outcome are presented in Supplementary Table 2.

**Table 1:** Details of studies and datasets used for analyses.

	**Traits**	**Samples size**	**Population**	**Consortium or cohort study (Link URL)**
Exposure	Serum magnesium	23,829	European	CHARGE and replication studies
Osteoporosis	Forearm BMD	53,236	European	GEFOS (http://www.gefos.org)
	Femoral neck BMD	53,236	European	
	Lumbar spine BMD	53,236	European	
	Heel BMD	426,824	European	GEFOS (http://www.gefos.org)
	Fracture	416,795	European	
Cardiometabolic diseases	Type 2 diabetes	898,130	European	DIAGRAM (http://diagram-consortium.org)
	Coronary artery disease	547,261	European	UK Biobank and CARDIoGRAMplusC4D (https://cvd.hugeamp.org/)
	Heart failure	977,323	European	UK Biobank (http://www.broadcvdi.org/)
	Atrial fibrillation	588,190	Mixed	Meta analysis of more than 50 studies (http://www.broadcvdi.org/)
Glycaemic traits	Fasting glucose	281,416	Mixed	MAGIC (https://magicinvestigators.org)
	Fasting insulin	281,416	Mixed	
	HbA1c	281,416	Mixed	

### Statistical Analyses

The exposure and outcome studies included in the two-sample MR study should not involve overlapping participants, but we could not estimate the degree of overlap based on the original articles. However, *F* statistic was used to estimate the bias from sample overlap, and this bias could be minimized by using strong instruments with *F* statistic >10 (38). The estimated F-statistics were relatively high (>30 for each SNP, Supplementary Table 1), and thus all SNPs were strong instruments.

To determine MR estimates of serum magnesium on each outcome, we conducted the inverse variance weighted (IVW) analysis because more than two SNPs were available. IVW method used a meta-analysis approach to combine Wald estimates for each SNP to get the overall estimates of the effect of serum magnesium concentrations on each outcome (39). The weighted median and MR–Egger regression methods were also applied to estimate the effects. Cochrane's *Q*-statistic was used to assess the heterogeneity of SNP effects, and *p* < 0.05 indicated significant heterogeneity (40).

Mendelian randomization pleiotropy residual sum and outlier test (MR-PRESSO) was used to assess the presence of pleiotropy, and the effect estimates were recalculated after outlying SNPs were excluded (41). The leave-one-out analysis was also conducted to assess the influence of individual variants on the observed associations (42). Furthermore, we performed the sensitivity analysis by removing potentially pleiotropic variants that were associated with confounding factors including calcium (rs11144134) and blood pressure (rs448378) and then studied the MR association between serum magnesium and outcomes.

The ethical approval for each study included in this investigation can be found in the original publications (including informed consent from each participant). The differences with *p* < 0.05 were considered statistically significant. In multiple testing, the adjusted *p*-value after Bonferroni correction (*p* < 0.05/4 = 0.0125) indicated a statistically significant difference. All of these analyses were conducted in R V.4.0.4 by using the R packages of “MendelianRandomization” (43), “TwoSampleMR,” (44) and “MR-PRESSO” (45).

## Results

### Osteoporosis

We evaluated the causal effect of serum magnesium on forearm BMD (Supplementary Figure 2A), femoral neck BMD (Supplementary Figure 2B), lumbar spine BMD (Supplementary Figure 2C), heel BMD (Supplementary Figure 2D), and fracture (Supplementary Figure 2E) in this MR analysis (Table 2). According to the primary (IVW) analysis, serum magnesium demonstrated no causal effect on forearm BMD (beta-estimate: −1.968, 95% CI: −4.397 to 0.461, SE: 1.239, *p* = 0.112), femoral neck BMD (beta-estimate: −1.171, 95% CI: −2.567 to 0.225, SE:0.712, *p* = 0.1), heel BMD (beta-estimate: −0.225, 95% CI: −0.365 to 0.914, SE:0.582, *p* = 0.698), or fracture (beta-estimate: 0.254, 95% CI: −0.946 to 1.453, SE:0.612, *p* = 0.679). However, genetically higher serum magnesium were inversely associated with lower lumbar spine BMD in the IVW analysis (beta-estimate: −1.982, 95% CI: −3.328 to −0.635, SE: 0.687, *p* = 0.004 < Bonferroni correction *p*), and this significant finding was also supported in weighted-median analysis (beta-estimate: −2.295, 95% CI: −3.739 to −0.851, SE: 0.737, *p* = 0.002 < Bonferroni correction *p*). Additionally, the weighted-median analysis also revealed the significant MR association between genetically high serum magnesium and low heel BMD (beta-estimate: −0.929, 95% CI: −1.376 to −0.481, SE: 0.228, *p* < 0.001).

**Table 2:** Mendelian randomization estimates of serum magnesium on osteoporosis.

**Variables**	**IVW**							**Weighted median**				**MR-Egger**							
	**Estimate**	**SE**	**95% CI**	**P-value**	**Q value**	**I^2^**	**Heterogeneity *P*-value**	**Estimate**	**SE**	**95% CI**	***P*** **-value**	**Estimate**	**SE**	**95% CI**	***P*** **-value**	**Intercept**	**SE**	**95% CI**	**Pleiotropy *P*-value**
Forearm BMD	−1.968	1.239	−4.397, 0.461	0.112	7.998	37.50%	0.156	−2.147	1.295	−4.686, 0.392	0.097	−4.366	4.051	−12.305, 3.573	0.281	0.018	0.029	−0.038, 0.074	0.531
Femoral neck BMD	−1.171	0.712	−2.567, 0.225	0.100	15.369	67.50%	0.009	−1.014	0.640	−2.268, 0.239	0.113	−3.051	1.906	−6.786, 0.684	0.109	0.012	0.011	−0.010, 0.033	0.289
Lumbar spine BMD	−1.982	0.687	−3.328, −0.635	0.004	8.021	37.70%	0.155	−2.295	0.737	−3.739, −0.851	0.002	−5.032	1.704	−8.372, −1.692	0.003	0.023	0.012	−0.001, 0.047	0.058
Heel BMD	−0.225	0.582	−0.365, 0.914	0.698	126.193	96.00%	<0.0001	−0.929	0.228	−1.376, −0.481	<0.001	−1.973	1.751	−5.405, 1.459	0.260	0.013	0.012	−0.011, 0.037	0.291
Fracture	0.254	0.612	−0.946, 1.453	0.679	11.070	54.80%	0.050	1.023	0.581	−0.115, 2.161	0.078	1.139	2.031	−2.841, 5.119	0.575	−0.007	0.014	−0.035, 0.021	0.644

### Cardiometabolic Diseases

This MR analysis included outcome measures of type 2 diabetes (Supplementary Figure 3A), coronary artery disease (Supplementary Figure 3B), heart failure (Supplementary Figure 3C), and atrial fibrillation (Supplementary Figure 3D). This primary (IVW) analysis found no obvious causal influence of serum magnesium on type 2 diabetes (beta-estimate: 0.405, 95% CI: −1.394 to 2.203, SE:0.918, *p* = 0.659), coronary artery disease (beta-estimate: −0.813, 95% CI: −1.826 to 0.201, SE: 0.517, *p* = 0.116), heart failure (beta-estimate: −0.461, 95% CI: −2.048 to 1.126, SE: 0.810, *p* = 0.569), or atrial fibrillation (beta-estimate: −1.297, 95% CI: −2.783 to 0.190, SE:0.759, *p* = 0.087, Table 3). These results were also confirmed by the weighted-median analyses.

**Table 3:** Mendelian randomization estimates of serum magnesium on cardiometabolic disease.

**Variables**	**IVW**							**Weighted median**				**MR-Egger**							
	**Estimate**	**SE**	**95% CI**	***P*** **-value**	***Q*** **-value**	***I*** **^2^**	**Heterogeneity *P*-value**	**Estimate**	**SE**	**95% CI**	***P*** **-value**	**Estimate**	**SE**	**95% CI**	***P*** **-value**	**Intercept**	**SE**	**95% CI**	**Pleiotropy *P*-value**
Type 2 diabetes	0.405	0.918	−1.394, 2.203	0.659	18.839	73.50%	0.002	−0.114	0.803	−1.687,1.459	0.887	4.462	2.288	−0.022, 8.946	0.051	−0.03	0.016	−0.062, 0.001	0.061
Coronary artery disease	−0.813	0.517	−1.826,0.201	0.116	10.383	51.80%	0.065	−0.419	0.471	−1.342, 0.504	0.374	1.831	1.069	−0.265, 3.927	0.087	−0.019	0.007	−0.034, −0.005	0.009
Heart failure	−0.461	0.810	−2.048, 1.126	0.569	12.976	61.50%	0.024	−0.751	0.682	−2.089, 0.585	0.270	−0.407	2.770	−5.836, 5.022	0.883	0.000	0.019	−0.038, 0.038	0.984
Atrial fibrillation	−1.297	0.759	−2.783, 0.190	0.087	13.670	63.40%	0.018	−1.125	0.606	−2.313, 0.062	0.063	0.455	2.419	−4.286, 5.195	0.851	−0.013	0.017	−0.046, 0.020	0.443
Fasting glucose	−0.096	0.233	−0.553, 0.362	0.682	16.865	70.40%	0.005	0.130	0.181	−0.225, 0.485	0.472	0.396	0.679	−0.935, 1.728	0.559	−0.004	0.005	−0.012, 0.005	0.438
Fasting insulin	0.188	0.178	−0.161, 0.538	0.291	7.815	36.00%	0.167	0.187	0.198	−0.202, 0.575	0.347	0.518	0.524	−0.510, 1.546	0.324	−0.002	0.004	−0.009,0.005	0.501
HbA1c	0.346	0.202	−0.049, 0.742	0.086	22.611	77.90%	<0.0001	0.526	0.155	0.222, 0.831	0.001	0.792	0.581	−0.346, 1.930	0.173	−0.003	0.004	−0.011, 0.004	0.411

### Glycaemic Traits

Fasting glucose (Supplementary Figure 3E), fasting insulin (Supplementary Figure 3F), and HbA1c (Supplementary Figure 3G) were involved to evaluate the glycaemic traits after the intervention of serum magnesium (Table 3). Based on the results of IVW analysis, serum magnesium showed no obvious MR association with fasting glucose (beta-estimate: −0.096, 95% CI: −0.533 to 0.362, SE: 0.233, *p* = 0.682), fasting insulin (beta-estimate: 0.188, 95% CI: 0.161–0.538, SE: 0.178, *p* = 0.291), or HbA1c (beta-estimate: 0.346, 95% CI: −0.049 to 0.742, SE: 0.202, *p* = 0.086). These results were confirmed by the weighted-median analyses except for HbA1c, because the positive finding between genetic serum magnesium and HbA1c was revealed by the weighted-median analyses (beta-estimate: 0.526, 95% CI: 0.222–0.831, SE: 0.155, *p* = 0.001).

### Power Estimation, Evaluation of Assumptions, and Sensitivity Analyses

The power to evaluate for causality in the observed association of serum magnesium with outcomes is shown in Supplementary Table 3, and all power measurements were 100%. Little evidence of directional pleiotropy was found for all models, except for coronary artery disease (MR–Egger intercept *p* = 0.009) (Tables 2, 3). Among the six SNP instrumental variables, the MR-PRESSO method identified one outlier (rs7965584) for femoral neck BMD, four outliers (rs4072037, rs7965584, rs3925584, and rs11144134) for heel BMD, one outlier (rs4072037) for type 2 diabetes, two outliers (rs4072037 and rs3925584) for fasting glucose and two outliers (rs4072037 and rs11144134) for HbA1c.

After excluding these outlying SNP variants, genetically higher serum magnesium displayed a significant causal role in lower femoral neck BMD (beta-estimate: −1.570, 95% CI: −2.770 to −0.371, SE: 0.612, *p* = 0.010 < Bonferroni correction *p*), whereas other MR associations were not changed (Table 4). The leave-one-out analysis demonstrated that the causal effect of serum magnesium concentrations on forearm BMD, femoral neck BMD, lumbar spine BMD, coronary artery disease, atrial fibrillation, and HbA1c was driven by potentially influential SNPs (Supplementary Figures 4, 5), and we should carefully interpret these results.

**Table 4:** Mendelian randomization estimates between serum magnesium and outcomes after excluding outliers detected by MR-PRESSO.

	**Estimate**	**SE**	**95% CI**	***P*** **-value**
Femoral neck BMD excluding one outlier (rs7965584)	−1.570	0.612	−2.770,0.371	0.010
Heel BMD excluding four outliers (rs4072037, rs7965584, rs3925584, rs11144134)	−0.188	0.289	−0.754,0.379	0.516
Type 2 diabetes excluding one outlier (rs4072037)	−1.174	0.609	−2.367,0.019	0.054
Fasting glucose excluding two outliers (rs4072037, rs3925584)	−0.067	0.243	−0.543,0.409	0.783
HbA1c excluding two outliers (rs4072037, rs11144134)	0.178	0.170	−0.154,0.511	0.293

Among the six instrumental variables, two confounding factors including calcium (rs11144134) and blood pressure (rs448378) may produce some influence on the MR analysis. After excluding these two SNPs, the significant inverse association between genetically serum magnesium and lumbar spine BMD was further confirmed (beta-estimate: −1.637, 95% CI: −3.076 to −0.197, SE: 0.734, *p* = 0.026, Table 4). Interestingly, a positive finding between genetically serum magnesium and HbA1c was also revealed after excluding these confounding factors (beta-estimate: 0.572, 95% CI: 0.348–0.797, SE: 0.114, *p* < 0.0001, Table 5).

**Table 5:** Mendelian randomization estimates between serum magnesium and outcomes after excluding potentially pleiotropic variants: rs11144134, rs448378.

	**Estimate**	**SE**	**95% CI**	***P*** **-value**
**Osteoporosis**				
Forearm BMD	−1.329	1.407	−4.087,1.428	0.345
Femoral neck BMD	−0.934	1.019	−2.931,1.064	0.360
Lumbar spine BMD	−1.637	0.734	−3.076,−0.197	0.026
Heel BMD	−0.068	0.808	−1.652,1.516	0.933
Fracture	−0.103	0.721	−1.517,1.310	0.886
**Cardiometabolic disease**				
Type 2 diabetes	0.614	1.276	−1.887,3.116	0.630
Coronary artery disease	−0.944	0.571	−2.062,0.175	0.098
Heart failure	−0.881	1.031	−2.902,1.140	0.393
Atrial fibrillation	−1.762	0.840	−3.408,−0.115	0.036
**Glycaemic traits**				
Fasting glucose	−0.039	0.338	−0.701,0.623	0.908
Fasting insulin	0.347	0.182	−0.011,0.704	0.057
HbA1c	0.572	0.114	0.348,0.797	0.000

## Discussion

Overall, our large multi-instrument approaches found the importantly causal effect of genetically increased serum magnesium on low lumbar spine BMD, and this inverse MR association was confirmed by various MR methods and sensitivity analyses. These positive findings indicated that high serum magnesium may be one of the significant risks of osteoporosis. In addition, we found no MR associations between serum magnesium and type 2 diabetes, coronary artery disease, heart failure and, atrial fibrillation.

The typical features of osteoporosis included low bone mass and microstructure deterioration of bone tissue, reduced BMD and bone strength, which may result in the increased risk of fracture (46–48). Animal and human experimental models demonstrated that Mg deficiency led to reduced osteoclastic and osteoblastic activity, osteopenia, and skeletal fragility (49, 50). Impaired bone growth and exacerbation of loss of bone mass were seen in the rat model with severely deficient Mg diets (51, 52). *In vitro* study revealed that a high concentration of Mg can promote the proliferation/differentiation behavior of osteoblasts (53). These suggested the potential benefits of high magnesium for osteoporosis.

Many observational studies have reported the association between serum magnesium and osteoporosis, but with conflicting results (54–58). Our MR study revealed that serum magnesium had no causal impact on forearm BMD, femoral neck BMD, heel BMD, or fracture. More interestingly, we found a significant MR association between high serum magnesium and low lumbar spine BMD, which was also confirmed by multiple sensitivity analyses. In addition, MR-PRESSO test found the causal role of high serum magnesium in low femoral neck BMD (beta-estimate: −1.570, 95% CI: −2.770 to −0.371, SE: 0.612, *p* = 0.010). The inverse MR association between high serum magnesium and low BMD was completely in contrast with the potential mechanisms of magnesium to affect osteoporosis, which included the promotion to bone mineralization, reduced function of osteoclasts, improved function of osteoblasts, and inducing the homeostasis of calcium by affecting parathyroid hormone (PTH) and 1,25(OH)2-vitamin D (1).

Several potential explanations may account for this inconsistency. First, serum magnesium concentrations may be not able to effectively reflect the total magnesium concentrations. Serum magnesium concentrations may remain relatively normal despite significant reductions of tissue and bone magnesium concentrations in patients with chronic latent magnesium deficiency, and serum magnesium concentrations may also underestimate the magnesium deficiency in healthy and ill people (1, 59). Secondly, low serum magnesium may predict the relatively high concentration of magnesium for bone metabolism in bone tissues. Thirdly, the concentration of magnesium may be required to be in a specific range called the “normal” physiological range for promoting bone formation and inhibiting bone resorption, and high magnesium may result in osteoporosis. This inverse association between serum magnesium and lumbar spine BMD was consistent with the inverse association between high serum calcium and low BMD (60). Fourthly, total serum magnesium and calcium remain relatively steady, and serum magnesium hemostasis is very complex, which is regulated by many factors such as PTH and 1,25(OH)2-vitamin D (54).

Regarding the association with serum magnesium concentrations and the risk of coronary artery disease, one previous MR study (61) included the same GWAS regarding serum magnesium concentrations (22) and one GWAS involving coronary artery disease with a smaller population of 1,84,305 individuals (62) than that population (5,47,261 individuals) included in our MR study (34). Contrary to the inverse association between serum magnesium concentrations and the risk of coronary artery disease concluded in the previous study (61), our MR analysis confirmed no MR association between them and did not find the benefits of high serum magnesium for preventing coronary artery disease. In addition, although one recent MR study [63] used the same summary genetic data about serum magnesium concentrations (22) and atrial fibrillation (36) compared with our MR analysis, three proxy SNPs for rs7965584, rs3925584, and rs448378 were used, whereas only one proxy SNP for rs7965584 was used in our MR analysis. Our results confirmed no MR association between genetically serum magnesium concentrations and atrial fibrillation, which was in contrast with their inverse association (63). We also found no causal association between serum magnesium concentrations and heart failure.

There are several important strengths in this study. The power to detect the causal association between serum magnesium and outcomes was sufficient because all power measurements were 100%. This two-sample MR study aims to investigate the causal effect of serum magnesium on osteoporosis and cardiometabolic diseases, and has the advantage of preventing reverse causation and potential confounding factors. The intercepts for the MR–Egger analysis suggest no directional pleiotropy except for coronary heart disease. Multiple sensitivity analyses are used to test the influence of pleiotropy on our causal estimate.

We also should consider several limitations. First, all the included participants were of predominantly European origin, and we could not directly recommend our findings for other populations. Second, the MR association between serum magnesium and each BMD (forearm BMD, femoral neck BMD, lumbar spine BMD, and heel BMD) were inconsistent and suggested that the causal effect of serum magnesium on BMD may act by the specific site. Third, lumbar spine BMD measurements have relatively higher standards errors compared with the other sites and may bias the results. Fourth, different techniques were used to measure serum magnesium concentrations, which may produce some heterogeneity.

## Conclusion

This two-sample MR study provides strong evidence to confirm that genetically raised serum magnesium is a significant causal risk factor of low lumbar spine BMD, which may provide new insights to prevent osteoporosis.

## Data Availability Statement

The original contributions presented in the study are included in the article/Supplementary Material, further inquiries can be directed to the corresponding author/s.

## Ethics Statement

The ethical approval for each study included in this investigation can be found in the original publications (including informed consent from each participant). The patients/participants provided their written informed consent to participate in this study.

## Author Contributions

BH, LX, JZ, LY, and MZ conducted study design, data collection, and statistical analysis. BH, LX, ZQ, YO, and WH conducted data interpretation, manuscript preparation, and literature search. BH conducted funds collection. All authors contributed to the article and approved the submitted version.

## Funding

This study was funded by the Foundation of the First Affiliated Hospital of Chongqing Medical University (PYJJ2018-13) and Natural Science Foundation of Chongqing (cstc2019jcyj-msxmX0836).

## Conflict of Interest

The authors declare that the research was conducted in the absence of any commercial or financial relationships that could be construed as a potential conflict of interest.

## Publisher's Note

All claims expressed in this article are solely those of the authors and do not necessarily represent those of their affiliated organizations, or those of the publisher, the editors and the reviewers. Any product that may be evaluated in this article, or claim that may be made by its manufacturer, is not guaranteed or endorsed by the publisher.
