# Supplementary material for: Causal Effect of Serum Magnesium on Osteoporosis and Cardiometabolic Diseases
Source: Front Nutr. 2021 Dec 3;8:738000. doi: 10.3389/fnut.2021.738000 (PMC8681341; doi:10.3389/fnut.2021.738000)
Supplement: Supplementary file 2 [file Data_Sheet_1.docx]

**Supplementary Figures**

**
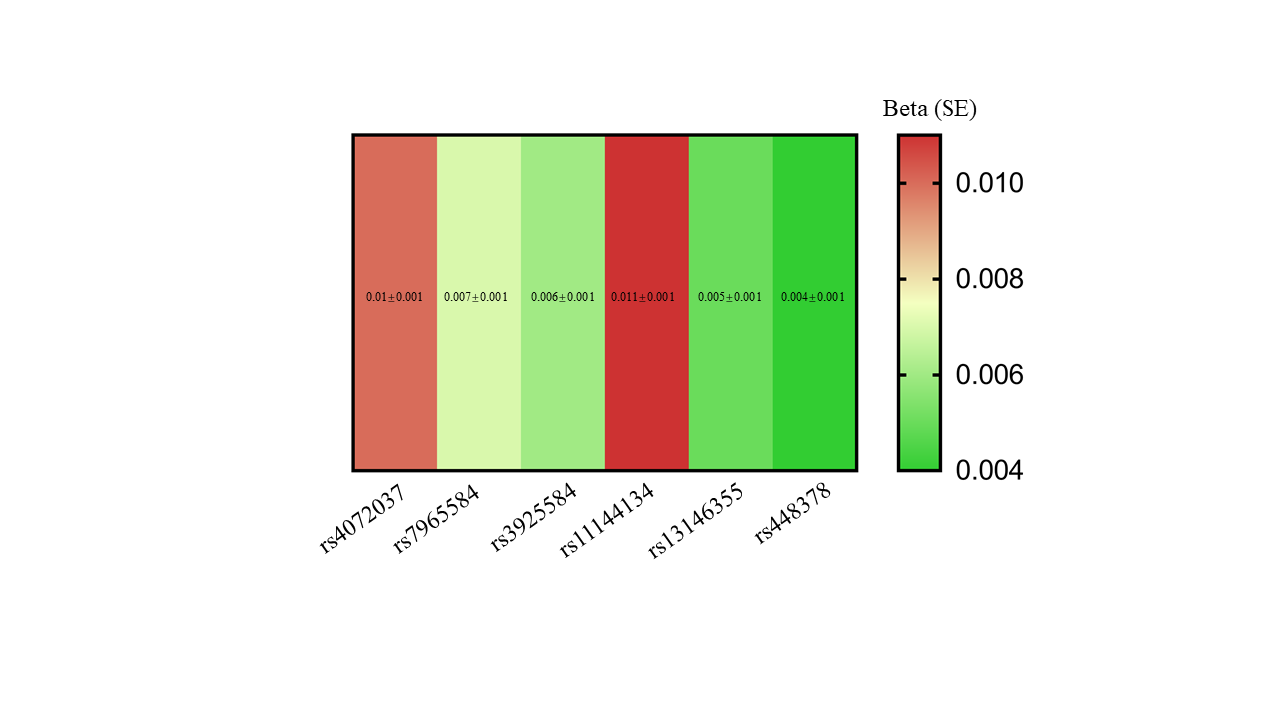
**

**Figure S1**

**
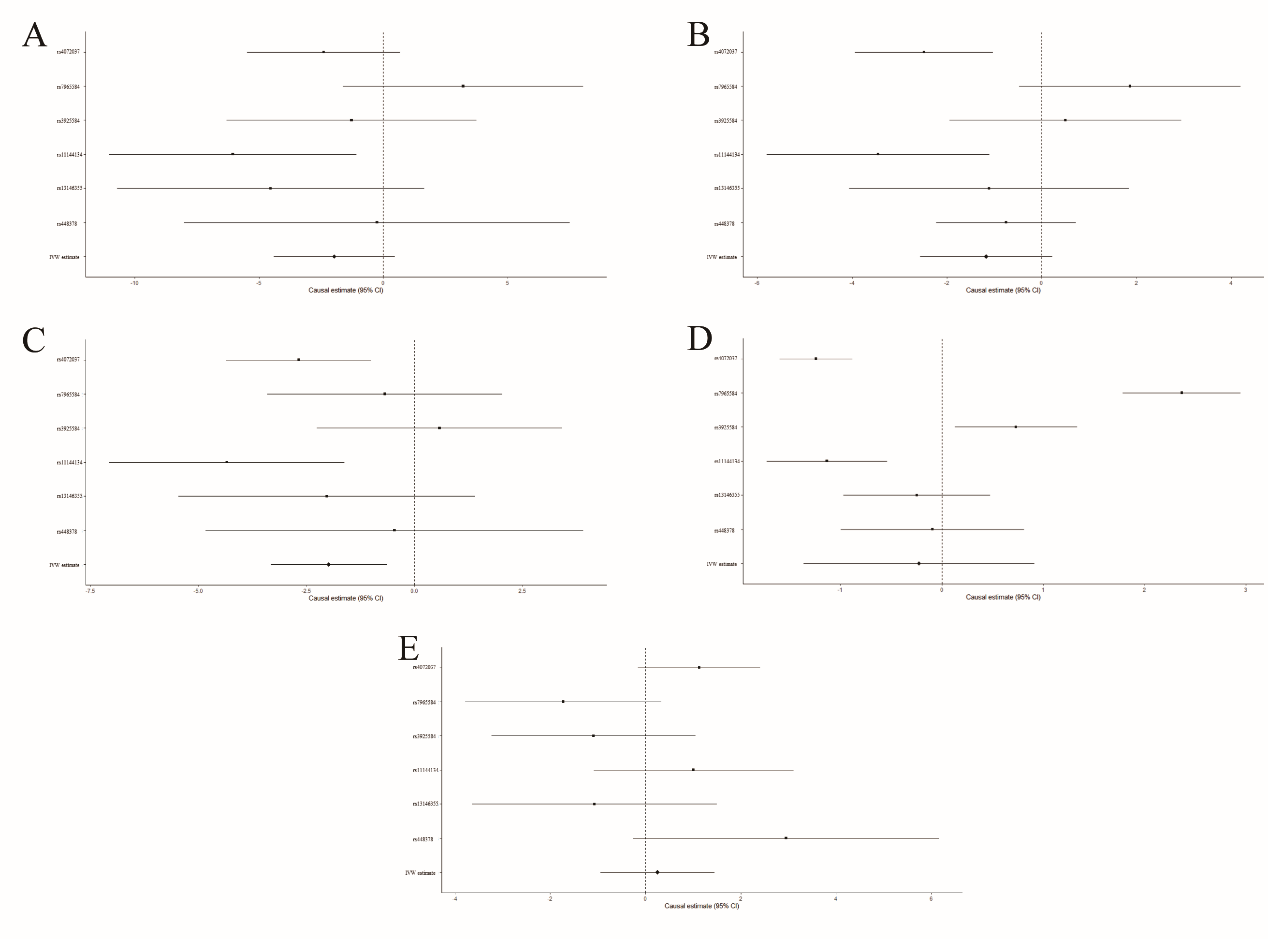
**

**Figure S2**

**
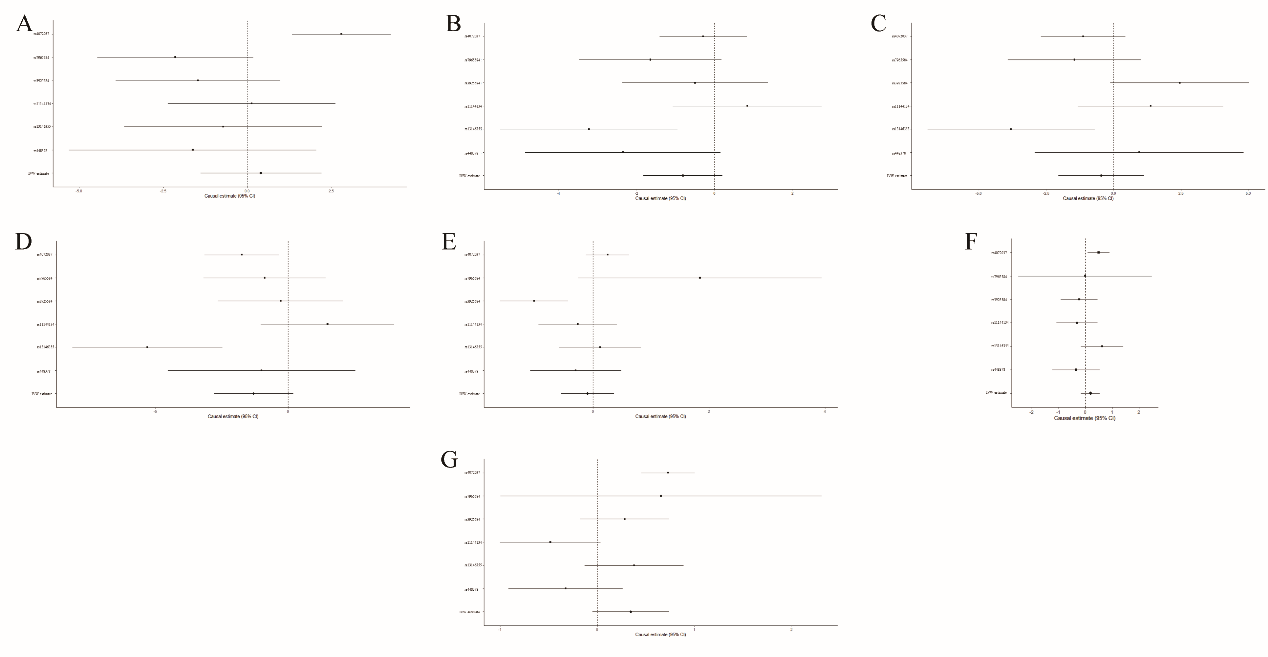
**

**Figure S3**

**
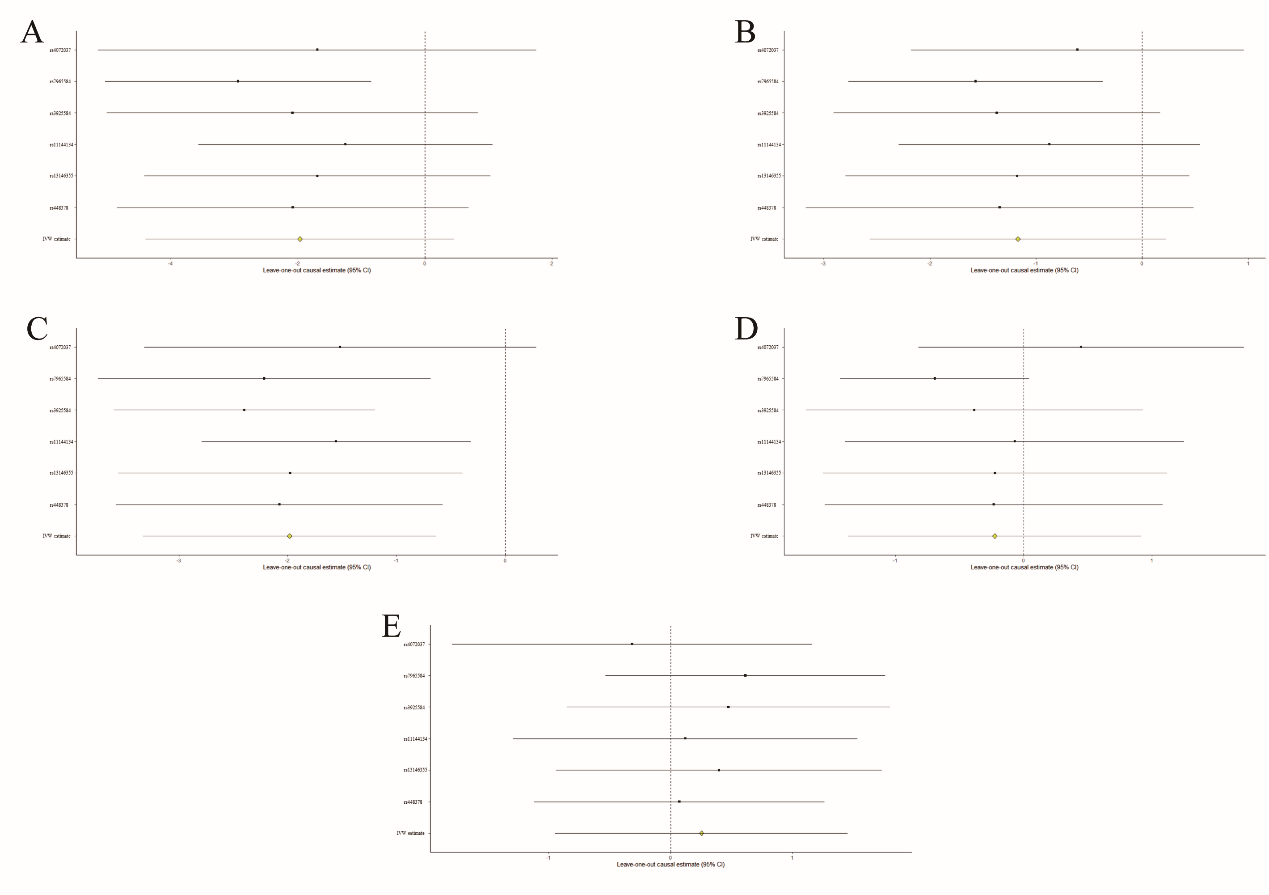
**

**Figure S4**

**
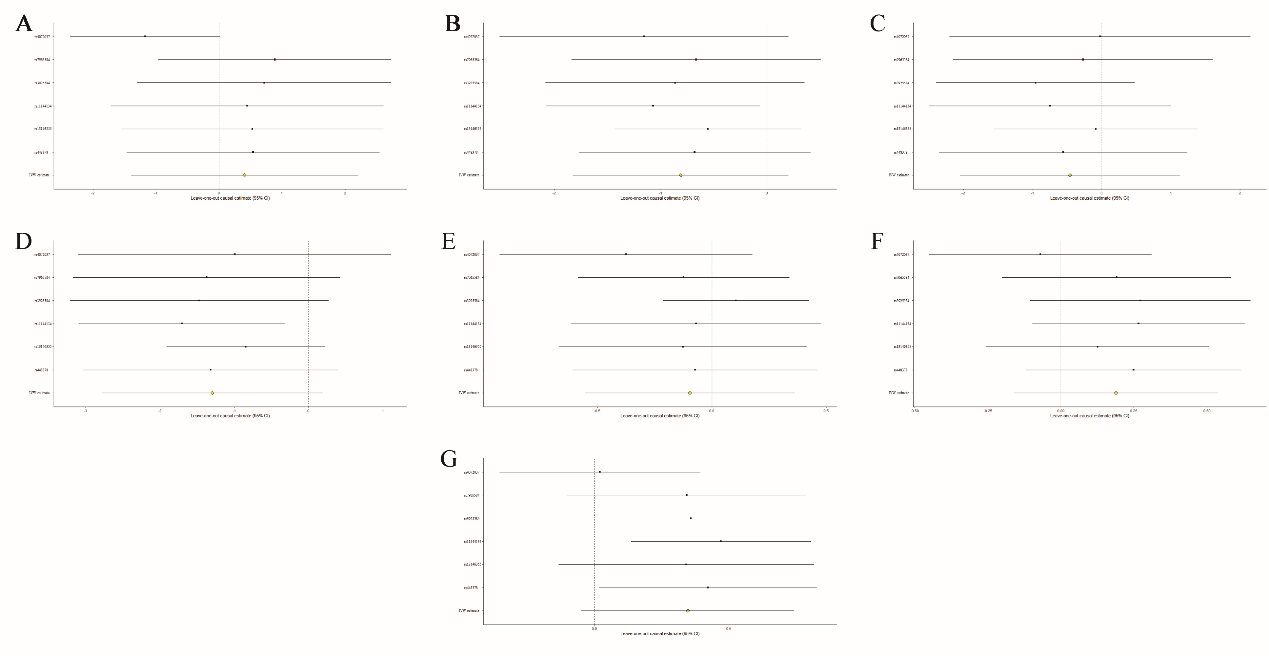
**

**Figure S5**

**Figure legend**

**Figure S1** Heat map showed the effects (beta and SE) of instrumental variables associated with serum magnesium.

**Figure S2** Causal influence of serum magnesium on forearm BMD (A), femoral neck BMD (B), lumbar spine BMD (C), heel BMD (D) and fracture (E).

**Figure S3** Causal influence of serum magnesium on type 2 diabetes (A), coronary artery disease (B), heart failure (C), atrial fibrillation (D), fasting glucose (E), fasting insulin (F) and HbA1c (G).

**Figure S4** Leave-one-out analysis for the causal effect of serum magnesium on forearm BMD (A), femoral neck BMD (B), lumbar spine BMD (C), heel BMD (D) and fracture (E).

**Figure S5** Leave-one-out analysis for the causal effect of serum magnesium on type 2 diabetes (A), coronary artery disease (B), heart failure (C), atrial fibrillation (D), fasting glucose (E), fasting insulin (F) and HbA1c (G).
